# Quercetin‐induced degradation of RhoC suppresses hepatocellular carcinoma invasion and metastasis

**DOI:** 10.1002/cam4.7082

**Published:** 2024-03-08

**Authors:** Chunlong Huang, Weihua Lai, Shuai Mao, Deli Song, Jihong Zhang, Xiao Xiao

**Affiliations:** ^1^ Department of Hepatobiliary Surgery, The first affiliated hospital Sun Yat‐Sen University Guangzhou Guangdong China; ^2^ Department of Pharmacy, Guangdong Provincial People's Hospital (Guangdong Academy of Medical Sciences) Southern Medical University Guangzhou Guangdong China; ^3^ Department of Pharmacology, Zhongshan School of Medicine Sun Yat‐sen University Guangzhou China

**Keywords:** hepatocellular carcinoma, metastasis, proteasomal degradation, quercetin, RhoC

## Abstract

**Background:**

Tumor metastasis and recurrence are major causes of mortality in patients with hepatocellular carcinoma (HCC) that is still lack of effective therapeutic targets and drugs. Previous reports implied that ras homolog family member C (RhoC) plays a toxic role on metastasis and proliferation of cancer.

**Methods:**

In this research, the correlation between RhoC and metastasis ability was confirmed by in vitro experiments and TCGA database. We explored whether quercetin could inhibit cell migration or invasion by transwell assay. Real‐time PCR, overexpression and ubiquitination assay, etc. were applied in mechanism study. Primary HCC cells and animal models including patient‐derived xenografts (PDXs) were employed to evaluate the anti‐metastasis effects of quercetin.

**Results:**

Clinical relevance and in vitro experiments further confirmed the level of RhoC was positively correlated with invasion and metastasis ability of HCC. Then we uncovered that quercetin could attenuate invasion and metastasis of HCC by downregulating RhoC's level in vitro, in vivo and PDXs. Furthermore, mechanistic investigations displayed quercetin hindered the E3 ligase expression of SMAD specific E3 ubiquitin protein ligase 2 (SMURF2) leading to enhancement of RhoC's ubiquitination and proteasomal degradation.

**Conclusions:**

Our research has revealed the novel mechanisms quercetin regulates degradation of RhoC level by targeting SMURF2 and identified quercetin may be a potential compound for HCC therapy.

## 1 INTRODUCTION

Liver cancer is estimated to be one of the most commonly diagnosed cancers and the leading cause of cancer death worldwide.^1^ Hepatocellular carcinoma (HCC) is the most common primary liver cancer type, followed by cholangiocarcinoma, and accounts for 70%–90% of primary liver cancer cases.^2^ Metastasis and recurrence are major contributors to the poor prognoses of HCC patients. Even though major advances have been made in terms of early diagnosis and therapy, the 5‐year survival rate and 5‐year disease‐free survival rate are still low.^3^, ^4^ The systemic therapy options available for HCC are limited and with poor prognosis.^5^ Even in recent years, the emergence of targeted therapy for liver cancer can prolong the average survival time of advanced liver cancer patients by about 3 months only.^6^ Therefore, it is essential to understand the mechanisms of HCC metastasis and search for drugs that prevent cancer progression.

Natural products were reported that made a great contribution to disease treatment, such as infectious and cancer diseases.^7^, ^8^ Quercetin, a Chinese herbal medicine‐derived compound, is an antianaphylactic used in America. It exhibits good safety and effectiveness. In addition, a previous study reported that quercetin could inhibit tumor growth and angiogenesis in vivo by regulating proliferation or apoptosis‐related genes.^9^ Here, we further discovered that quercetin attenuates the metastasis and recurrence of HCC by promoting ras homolog family member C (RhoC) ubiquitination.

RhoC is a small signaling G protein in the superfamily of GTP enzymes renin, which has endogenous GTP enzyme activity. RhoC can be converted between inactive GDP binding forms and active GTP binding forms.^10^, ^11^ In the GTP‐binding form, Rho GTPases bind to downstream molecules such as ROCK, PIP2 and p140mDia, which leads to cytoskeletal rearrangement, the assembly of actin and myosin filaments, and the formation of adhesive plaque complexes.^12^, ^13^, ^14^ Previous studies have provided clues that RhoC plays a critical regulatory role in cell migration and invasion.^15^ Here, it was further validated that RhoC shows a positive association with the metastasis (invasion or migration) ability of HCC according to in vitro and in vivo experiments and clinical relevance analysis. Quercetin is first reported to enhance the proteasomal degradation of RhoC, resulting in the inhibition of cell migration and invasion in HCC. Our findings provided a rationale supporting quercetin as a potential therapeutic medicine for HCC treatment.

## 2 METHODS

### 2.1 Reagents and antibodies

Cycloheximide (CHX), MG132 and quercetin were purchased from Sigma‐Aldrich (USA). Quercetin was dissolved in DMSO and then diluted in DMEM in vitro. In in vivo experiments, the solvent for quercetin was 25% hydroxypropyl‐β‐cyclodextrin.^16^, ^17^ Anti‐RhoC and SMAD specific E3 ubiquitin protein ligase 2 (SMURF2) were from Abcam (UK). Anti‐NEDD4 E3 ubiquitin protein ligase (NEDD4) and anti‐GAPDH antibodies were obtained from Cell Signaling Technology (USA). The anti‐WW domain containing E3 ubiquitin protein ligase 1 (WWP1) antibody was purchased from Proteintech (China).

### 2.2 Cell culture and tissue specimens

The HCC cell lines (Huh‐7, Li‐7 and SK‐HEP‐1) were bought from the Institute of Biochemistry and Cell Biology of the Chinese Academy of Sciences (Shanghai, China). Normal human hepatocyte (HH) cells were from ScienCell Research Laboratories (USA). Additional HCC cell lines (Hep3B, PLC, and HepG2) and 293 T were obtained from the American Type Tissue Culture Collection (ATCC; USA). The cells were maintained in DMEM (Gibco, USA) supplemented with 10% fetal bovine serum (FBS, Gibco) and 1% penicillin/streptomycin. The passage number of the cell lines was <20.

HCC tissues were obtained from the First Affiliated Hospital of Sun Yat‐Sen University. All cases were histologically confirmed. Written informed consent was obtained from each patient, and the study was approved by the Institute Research Medical Ethics Committee of the First Affiliated Hospital, Sun Yat‐Sen University.

### 2.3 Transwell assay

The migration and invasion assays were performed using Transwell chambers with filters (Corning, USA) as described.^18^ The filters applied for invasion assays were coated with Matrigel (BD Bioscience, USA) diluted at a ratio of 1:6 with serum‐free DMEM. Cells in serum‐free DMEM were added to the upper chambers. Then, DMEM supplemented with 10% FBS was added to the lower chambers as a chemoattractant. After quercetin incubation for 24 h, cells on the upper membrane surface were wiped off. The cells that invaded across the Matrigel membrane were fixed with 4% paraformaldehyde and stained with 0.1% crystal violet. The invaded cells were counted (in five randomly selected fields for each membrane) under a light microscope. The filters used for the migration assays were not coated with Matrigel.

### 2.4 Ubiquitination assay

293T cells were planted at 100 mm cell culture dishes and maintained in Dulbecco's modified Eagle's medium with 10% FBS. Following the Transfection Reagent instructions, the Flag‐RhoC overexpression plasmids were transfected into 293T cells using Lipofectamine 3000 (Thermo, USA). After 48 h, the cells were treated with 20 μM MG132 for 6 h and then collected to purify the RhoC protein. The polyubiquitinated RhoC protein from the cell lysate was separated on SDS‐polyacrylamide gels, transferred to PVDF membranes and blotted with anti‐ubiquitin antibody (Cell Signaling Technology).^19^

### 2.5 Animal models

Four‐week‐old male nude mice were purchased from Guangdong Medical Laboratory Animal Center. All experimental procedures involving animals were approved by the Animal Ethical and Welfare Committee of Sun Yat‐Sen University (China). Our research was performed in agreement with the Declaration of Helsinki. At the end of the experiments, the mice were euthanized by isoflurane overdose, followed by cervical dislocation.

For the subcutaneous model, 1 × 10^7^ Huh7 cells were inoculated subcutaneously into the hind flanks of mice. When palpable tumors developed (~50 mm^3^), the mice were randomly divided into vehicle/quercetin groups (*n* = 5 each). Quercetin was administered by intraperitoneal injection at 50 mg/kg for 21 consecutive days. Tumor length and width were measured, and then the volume was calculated according to the formula: (length × width^2^) × 0.5.

For the orthotopic implantation model, Huh7 cells (6 × 10^6^) were inoculated subcutaneously into the dorsal flanks of the nude mice. After 3 weeks, tumor tissue was collected from tumor‐bearing mice and cut into pieces (~8 mm^3^). The tissue blocks were implanted into the livers of nude mice. The mice were anesthetized with isoflurane when the tissues blocks were implanted. Male nude mice (*n = 5* animals per group) were randomly distributed among the following experimental groups: the vehicle‐injected control group or the quercetin‐injected group (50 mg/(kg d)). Mice received intraperitoneal injection for three consecutive weeks. The isolated tumors were fixed in 10% formalin and subsequently paraffin embedded.

For the patient‐derived xenografts (PDXs), fresh tumor samples were from the First Affiliated Hospital of Sun Yat‐sen University. Tumor fragments (2 × 1 × 1 mm^3^) were surgically xenografted into the right flanks of male NSG mice (4–5 weeks) under isoflurane anesthesia. When the tumor size reached almost 100 mm^3^, the mice were randomly assigned to two groups (*n* = 6): the vehicle‐injected control group and the group administered quercetin at a dose of 50 mg/(kg d). The mice in the vehicle or treatment group were injected intraperitoneally for three consecutive weeks.

### 2.6 Western blotting

Cells were lysed on ice with RIPA lysis buffer and protease inhibitor mixture cocktail (Roche, Switzerland). Equal amounts of protein were separated by SDS‐polyacrylamide gel (Bio‐Rad, USA) electrophoresis and transferred onto PVDF membranes. After blocking, the membranes were incubated with primary antibodies and then with the appropriate secondary HRP‐conjugated antibody. The immunoreactive bands on the blots were visualized with an enhanced chemiluminescence reagent kit (Bio‐Rad, USA), and the intensities were quantified using ImageJ software.

### 2.7 Reverse‐transcription quantitative real‐time polymerase chain reaction (RT‐qPCR)

Total RNA was extracted with an ultrapure RNA extraction kit (CW Biotech, Beijing, China). Then, reverse transcription was performed from 1 μg of total RNA using a Revert Aid First Strand cDNA Synthesis Kit (Thermo Scientific, USA) according to the manufacturer's recommendation. RT‐qPCR was performed with TB Green™ Premix Ex Taq™ II (TaKaRa, Japan) with an Applied Biosystems 7500 Fast Real‐Time PCR System (Life Technologies). The mRNA level was calculated according to the 2^−(ΔΔCt)^ method and then normalized to β‐actin. The amplification primers (Sangon, China) were as follows (5′ to 3′): RhoC (forward, CCTGAGGCAAGACGAGCAC; reverse, GATCCGGTTCGCCATGTCC), SMURF1 (forward, ATTCGATAACCATTAGCGTGTGG; reverse, CGCCGGTTCCTATTCTGTCTC), SMURF2 (forward, CGG‐ TTGTGTTCGTCTTCTTTCC; reverse, GCCCGAGTTTGCATAAATCCA), NEDD4 (forward, TCCAATGATCTAGGGCCTTTACC; reverse, CCAA‐CCGAGGATCTTCCCAT), UBE3C (forward, TGGCCCCAACCTTACCCTT; reverse, GCAGCAACCTGCAACAGAG), CBL (forward, TGGTGCG‐ GTTGTGTCAGAAC; reverse, GGTAGGTATCTGGTAGCAGGTC), UBR5 (forward, CCAGACAGATTGGAATTGGGTAA; reverse, CATGGAGAG‐ TCGCTTGTCCT), WWP1 (forward, AGAACTGGTTCGGAACAGCAA; reverse, TTAAAGTGCGATGGCTCCAAA), WWP2 (forward, CAAAGCCCAAGGTGCATAATCG; reverse, CCAATGCGCTTCCCAGTCT), β‐actin (forward, GATCATTGCTCCTCCTGAGC; reverse, ACTCCTGCTTGCTGATCCAC), SYTL4 (forward, CGCTTTGCTTACCGCA‐ CAG; reverse, GCTATCCAGACTCTCACTCTCA), and HUWE1 (forward, TGCCAGTGCTTGTAAGGAACT; reverse, TGGTGACAAATGTTATCTGGT‐ CC).

### 2.8 Immunohistochemistry (IHC)

The expression levels of RhoC and SMURF2 in the tumors were assessed by immunohistochemistry (IHC). Briefly, tumor slides (4 μm thick) were dewaxed in xylene, dehydrated in a graded ethanol series and immersed in 0.3% H_2_O_2_‐methanol to eliminate the endogenous peroxidase activity. Then slides were washed with PBS and probed with primary antibody at 4°C overnight. After being washed, the sections were incubated with horseradish peroxidase‐conjugated secondary antibody at room temperature for 2 h. Immunostaining was visualized with diaminobenzidine, and the sections were counterstained with hematoxylin.^20^

### 2.9 Cell viability assays

Cells were seeded in 96‐well plates in 0.1 mL medium. After treatment, cell counting kit 8 (CCK8) assay (beyotime) was added to the cells, and cells were allowed to grow at 37°C for 2 h. Then the optical absorbance was measured at 450 nm using a microplate reader (iMark, Bio‐Rad).

### 2.10 Statistics

All statistical analyses were performed using GraphPad Prism 8.0 software, and all error bars indicated the standard deviation (SD). The statistical significance of differences between the two groups was determined by a two‐tailed Student's *t*‐test, and one‐way ANOVA was applied for comparisons between multiple groups. The Pearson correlation coefficient was applied to calculate statistical dependence. The tumor volume was analyzed by repeated‐measures one‐way ANOVA. Differences were considered significant when the *p* < 0.05.^21^ All experiments were finished at least three times.

## 3 RESULTS

### 3.1 The expression of RhoC may be positively associated with HCC invasion and metastasis

**FIGURE 1:**
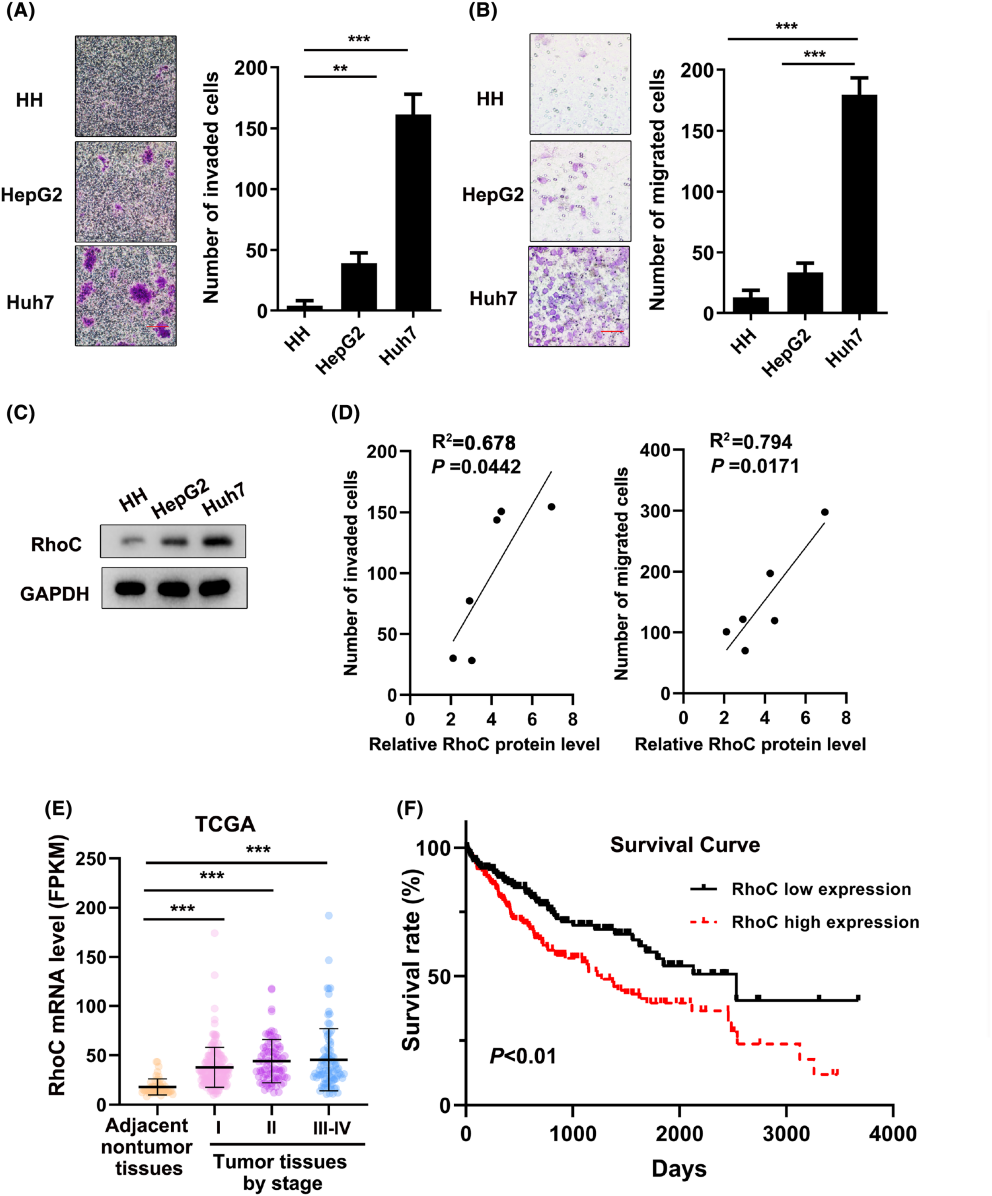
The RhoC level is related to HCC cell invasiveness and migration. (A) Detection of the invasive ability of cell lines. Cells were seeded in the upper chambers. Then invaded cells were counted 24 h later. (B) Detection of the migration ability of cell lines. Cells are seeded in the upper chambers. The migrated cells were counted 24 h later. Scale bars: 50 μm. (C) The expression of RhoC protein in cell lines was tested by western blotting. GAPDH was used as an internal control. (D) The number of invaded or migrated in HCC cells was counted. HCC cell lines: Li‐7, PLC, HepG2, SK‐HP‐1, Hep3B, and Huh7. (E) Expression levels of RhoC in HCC tissues of different stages (stage I, *n* = 171; stage II, *n* = 34; stage III‐IV, *n* = 90) and adjacent non‐tumor tissues (*n* = 50). F, Kaplan–Meier analysis of the survival of HCC patients with high or low RhoC levels using the log‐rank test. **p < 0.01, ***p < 0.001.

The invasion and migration mainly depend on cancer cell motility and are indispensable for tumor metastasis.^22^ To evaluate cell migration and invasion, Transwell assays were performed with different cell lines. The results showed that the cell invasiveness was the lowest in HH cells, moderate in HepG2 (nonmetastatic hepatoma) cells and the highest in Huh7 (metastatic hepatoma) cells (Figure 1A). The cell migration trend was consistent with the cell invasion trend (Figure 1B).

To further study the protein expression of RhoC in these HCC cell lines, western blotting was performed. The data implied that with the increase in cell aggressiveness (HH < HepG2 < Huh7), RhoC protein levels were also remarkably increased (Figure 1C). Subsequently, the number of invasive cells and the relative RhoC protein level were detected in a series of HCC cell lines. Analysis of cell lines revealed a positive correlation between metastasis ability (invasion/migration) and RhoC expression (Figure 1D). Above all, our findings suggest that cell invasion ability might be positively correlated with RhoC protein expression.

Moreover, we collected available data from The Cancer Genome Atlas (TCGA) and analyzed the expression of RhoC in tumor tissues of different tumor‐node metastasis (TNM) stages and adjacent nontumor tissues.^23^ The mRNA levels of RhoC were positively associated with the TNM stages of tumors and were significantly higher in tumor tissues of all stages than in adjacent nontumor tissues (Figure 1E). Additionally, the data from the TCGA also indicated that high RhoC levels were significantly associated with a poor survival rate (Figure 1F). Therefore, our data supported the notion that RhoC is highly expressed in HCC and correlated with the malignancy and metastasis ability of HCC.

### 3.2 Quercetin inhibits the invasion and migration of HCC cells in a concentration‐ and time‐dependent manner

**FIGURE 2:**
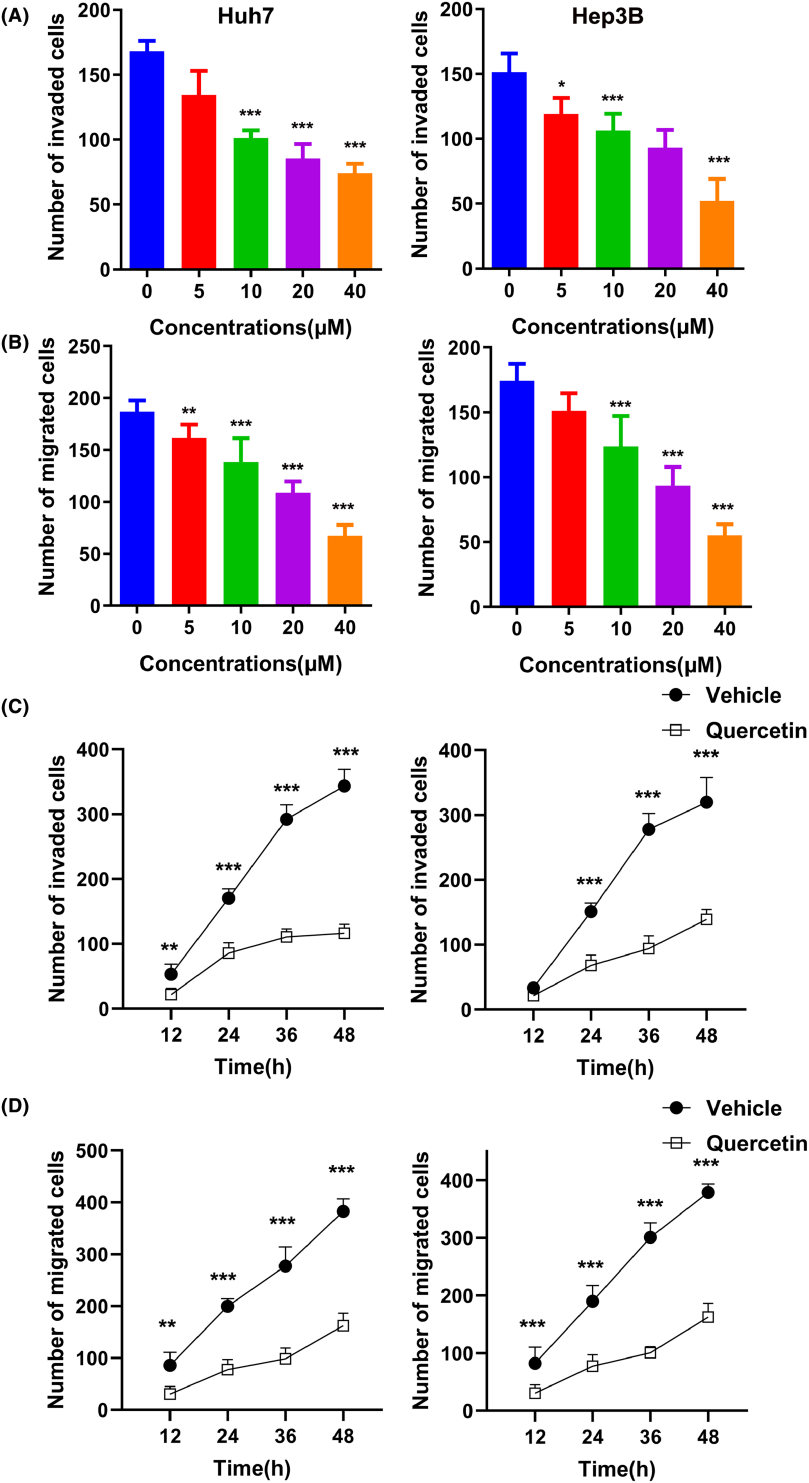
Quercetin inhibits the invasion and migration of HCC cells. Huh7 (left) and Hep3B (right) cells were treated with different concentrations of quercetin (0, 5, 10, 20 or 40 μM) for 24 h. (A) Quercetin reduced cell invasion in a concentration‐dependent manner. (B) Quercetin decreased cell migration in a concentration‐dependent manner. Huh7 (left) and Hep3B (right) cells were incubated with quercetin (20 μM) at the indicated times. (C) Quercetin suppressed cell invasion at various times. (D) Quercetin reduced cell migration at different times. All are compared with respective control groups. *p < 0.05, **p < 0.01, ***p < 0.001.

It is urgent to search for effective drugs to combat the recurrence and metastasis of HCC.^24^ In the present study, we found that quercetin reduced the invasive and migration abilities of HCC cells (Figure S1), but did not significantly decrease cell viability (Figure S2). The RhoC protein level in Hep3B and Huh7 is higher than other cell lines. Therefore, Hep3B and Huh7 were used in subsequent research. Huh7 and Hep3B cells were incubated with quercetin at varying concentrations (0, 5, 10, 20, and 40 μM), and then the numbers of invaded and migrated cells were counted. Notably, quercetin decreased the numbers of invaded cells (Figure 2A, Figure S3A) and migrated cells (Figure 2B, Figure S3B) in a dose‐dependent manner. Therefore, quercetin decreases the cell invasion and migration abilities of HCC in a concentration‐dependent way.

Moreover, our data showed that the invasion of vehicle‐treated cells was markedly faster than that of quercetin‐treated cells (Figure 2C). Consistent with these results, quercetin treatment obviously hindered cell migration over time (Figure 2D). Hence, quercetin slows the invasion and migration progression of HCC cells. The epithelial‐mesenchymal transition (EMT) is a crucial developmental program that is often activated during cancer invasion and metastasis.^25^ Here we discovered that quercetin downregulated pro‐EMT markers N‐Cadherin and Vimentin, and upregulated anti‐EMT protein E‐cadherin, which suggests quercetin could inhibit EMT of HCC cells (Figure S4).

### 3.3 Quercetin suppresses HCC cell invasion and migration by targeting RhoC

**FIGURE 3:**
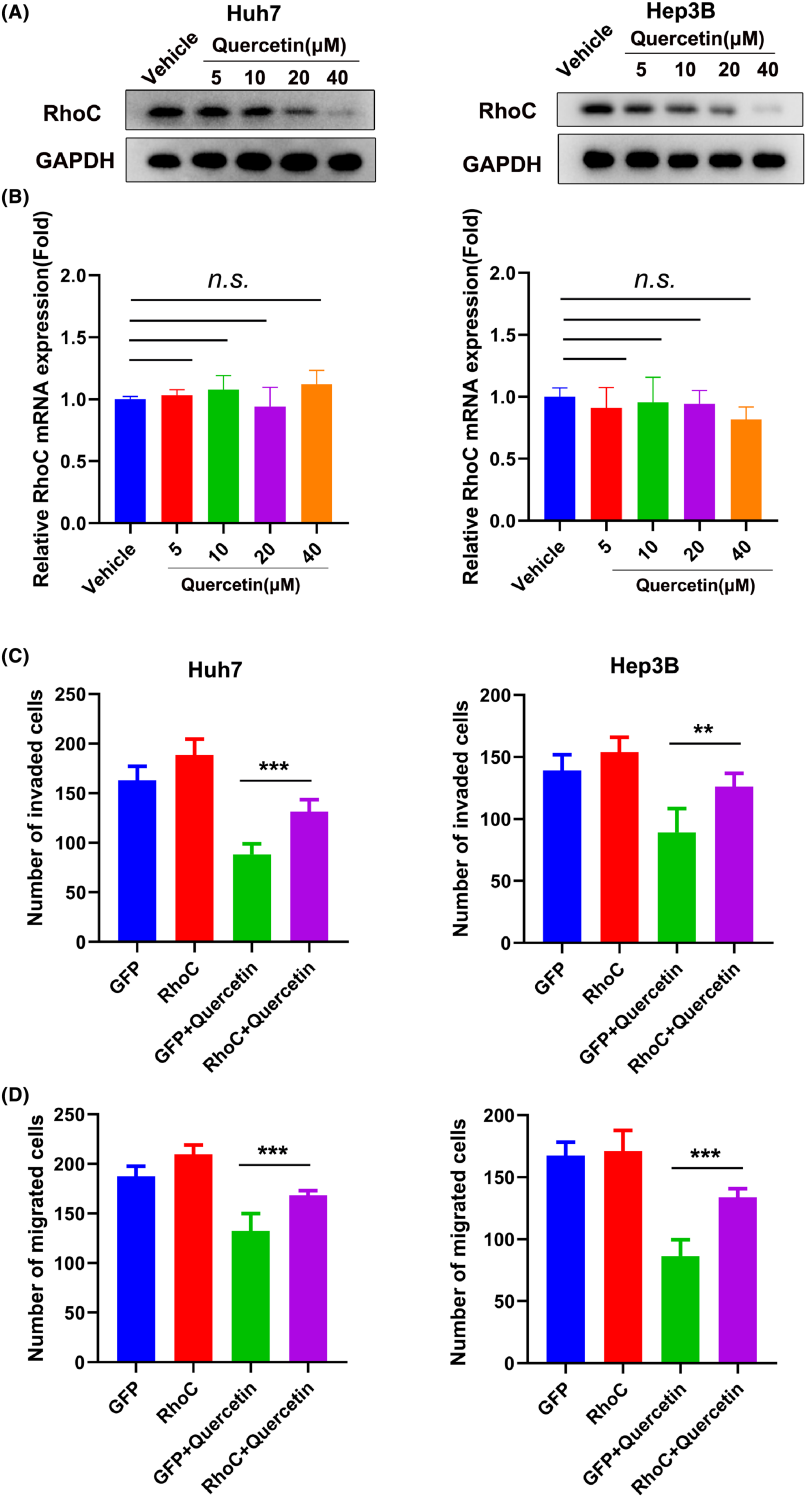
Quercetin suppresses RhoC levels without affecting mRNA expression. (A) Huh7 (left) and Hep3B (right) cells were incubated with various concentrations of quercetin. The cells were harvested and lysed after 24 h, and the expression of RhoC was determined by western blotting. GAPDH was used as an internal control. (B) RT‐qPCR was used to assess the mRNA expression of RhoC in the quercetin‐treated and vehicle groups. (C) Huh7 (left) and Hep3B (right) cells were transfected with plasmids expressing GFP (negative control) or RhoC and then incubated with quercetin. RhoC overexpression promoted the invasion of cells treated with quercetin. (D) The overexpression of RhoC enhanced the migration ability of quercetin‐treated cells. **p < 0.01, ***p < 0.001.

Interestingly, the western blotting results indicated that quercetin lowers the protein level of RhoC. After Huh7 and Hep3B cells were cultured with different concentrations of quercetin. In contrast with 0 μM group, RhoC protein expression notably decreased at concentration of 40 μM (Figure 3A). However, the RT‐qPCR results displayed no significant difference between the quercetin‐treated and vehicle‐treated groups (Figure 3B). Taken together, the above results validated that quercetin suppresses RhoC protein expression but not RhoC mRNA expression in a concentration‐dependent manner.

Next, we further investigated whether quercetin inhibits cell invasion and migration by targeting RhoC. Remarkably, quercetin treatment decreased the number of invasive cells, and this effect was reversed by RhoC overexpression (Figure 3C). Consistently, overexpression of RhoC attenuated the inhibitory effect of quercetin on cell migration (Figure 3D). Moreover, knockdown of RhoC promoted anticancer effect of quercetin on cell invasion and migration (Figure S5). Hence, our data verified that quercetin hinders cell invasion and migration of HCC by downregulating RhoC.

### 3.4 Quercetin accelerates RhoC degradation via the proteasome pathway

**FIGURE 4:**
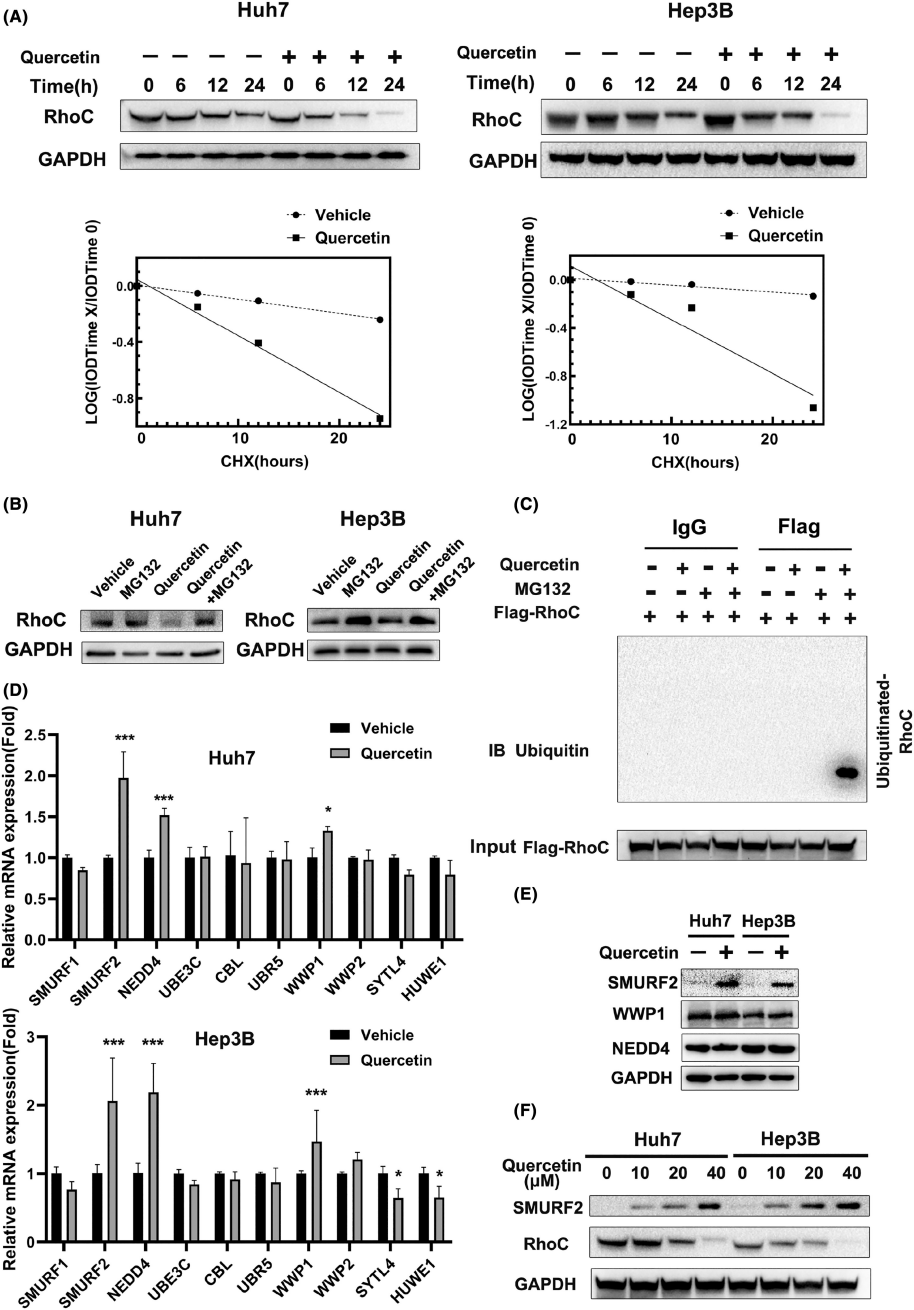
Quercetin enhances RhoC degradation through the proteasomal pathway. (A) Quercetin decreased the turnover rate of RhoC in Huh7 (left) or Hep3B (right) cells. Vehicle‐ and quercetin‐treated cells were exposed to 100 μg/mL CHX, a protein synthesis inhibitor. Cells were collected at the indicated times (0–24 h) after treatment and detected by western blotting for RhoC and GAPDH. (B) Loss of RhoC in response to quercetin was mediated by proteasomal degradation. Vehicle/quercetin‐treated cells were cultured with 20 μM MG132 for 6 h. The cells were harvested and lysed, and the whole‐cell lysates were subjected to western blotting for RhoC and GAPDH. (C) Quercetin increased the ubiquitination level of RhoC compared to that in the control group of 293T cells (after MG132 enrichment for ubiquitination). Cells were treated with the indicated plasmids and quercetin and then incubated with MG132 for 6 h or not. Ubiquitinated RhoC was detected by immunoprecipitation and western blotting assays. IB, Immunoblotting. (D) Quercetin promoted the mRNA expression of E3 ligases related to RhoC (SMURF2, NEDD4, and WWP1) in Huh7 and Hep3B cells. Cells were collected for total mRNA extraction after quercetin treatment. qRT‐PCR was performed to determine the changes in the expression of the indicated genes (*n* ≥ 4, *p < 0.05, ***p < 0.001). (E) Quercetin increased the protein level of SMURF2 but not that of WWP1 or NEDD4. Cells were incubated with quercetin or vehicle and harvested at 24 h for western blot analysis. (F) Quercetin upregulated SMURF2 expression and reduced the protein level of RhoC in a dose‐dependent manner.

To further explore the mechanism by which quercetin decreases the protein level of RhoC, CHX chase experiments were conducted to evaluate RhoC stability in HCC cells. CHX is a protein biosynthesis inhibitor.^26^ The of RhoC protein in quercetin treatment has a fast turnover rate comparing with the vehicle group, suggesting that quercetin downregulates RhoC protein level by controlling RhoC's post‐translational stability (Figure 4A). In addition, to assess whether RhoC protein stability is attenuated by quercetin via the proteasome pathway, Huh7 and Hep3B cells were incubated with MG132 after quercetin treatment. Notably, MG132 partly restored RhoC levels in cells treated with quercetin (Figure 4B). Moreover, western blot analysis indicated that RhoC was highly ubiquitinated in the quercetin treatment group compared with control groups (Figure 4C). These observations suggested that quercetin promotes the degradation of RhoC through the proteasome pathway.

Protein ubiquitination is a posttranslational modification that is essential for regulating various biological processes.^27^ Ubiquitination is catalyzed and mediated by three main enzymes: E1 ubiquitin activating enzymes, E2 ubiquitin conjugating enzymes, and E3 ubiquitin ligases. It is well known that E3 ligases govern the efficiency and substrate specificity of the ubiquitination reaction.^28^, ^29^ Based on these considerations, we suspected that quercetin enhances the degradation of RhoC by increasing expression of E3 ligases. We used RhoC as a substrate to predict its ubiquitin ligases by employing the UbiBrowser database (http://ubibrowser.ncpsb.org/ubibrowser/).^30^ SMURF1, SMURF2, NEDD4, UBE3C, CBL, UBR5, WWP1, WWP2, SYTL4, and HUWE1 were selected from the UbiBrowser database as potential ligases targeting RhoC (Table S1). To identify the E3 ligases involved in the degradation of RhoC, their mRNA levels were evaluated after quercetin treatment. Quercetin significantly raised the mRNA expression of SMURF2, NEDD4, and WWP1 in Huh7 and Hep3B cells (Figure 4D). Considering these results, the protein levels of SMURF2, NEDD4, and WWP1 were further examined by western blotting after quercetin treatment. A remarkable upregulation of SMURF2 protein was observed in the quercetin treatment group versus the control group at the indicated times (Figure 4E). Additionally, quercetin increased the SMURF2 protein level and decreased the RhoC level (Figure 4F). In order to determine if SMURF2 regulates RhoC protein levels, the ectopic expression or knockdown of SMURF2 experiments were performed in HCC cells. These evidences supported that SMURF2 is a negative regulator of RhoC protein (Figure S6). Our observations proved that SMURF2 participates in quercetin‐mediated RhoC degradation. Moreover, the interaction RhoC with SMURF2 is identified by Co‐IP assay (Figure S7).

### 3.5 Quercetin represses the growth and metastasis of HCC in vivo

**FIGURE 5:**
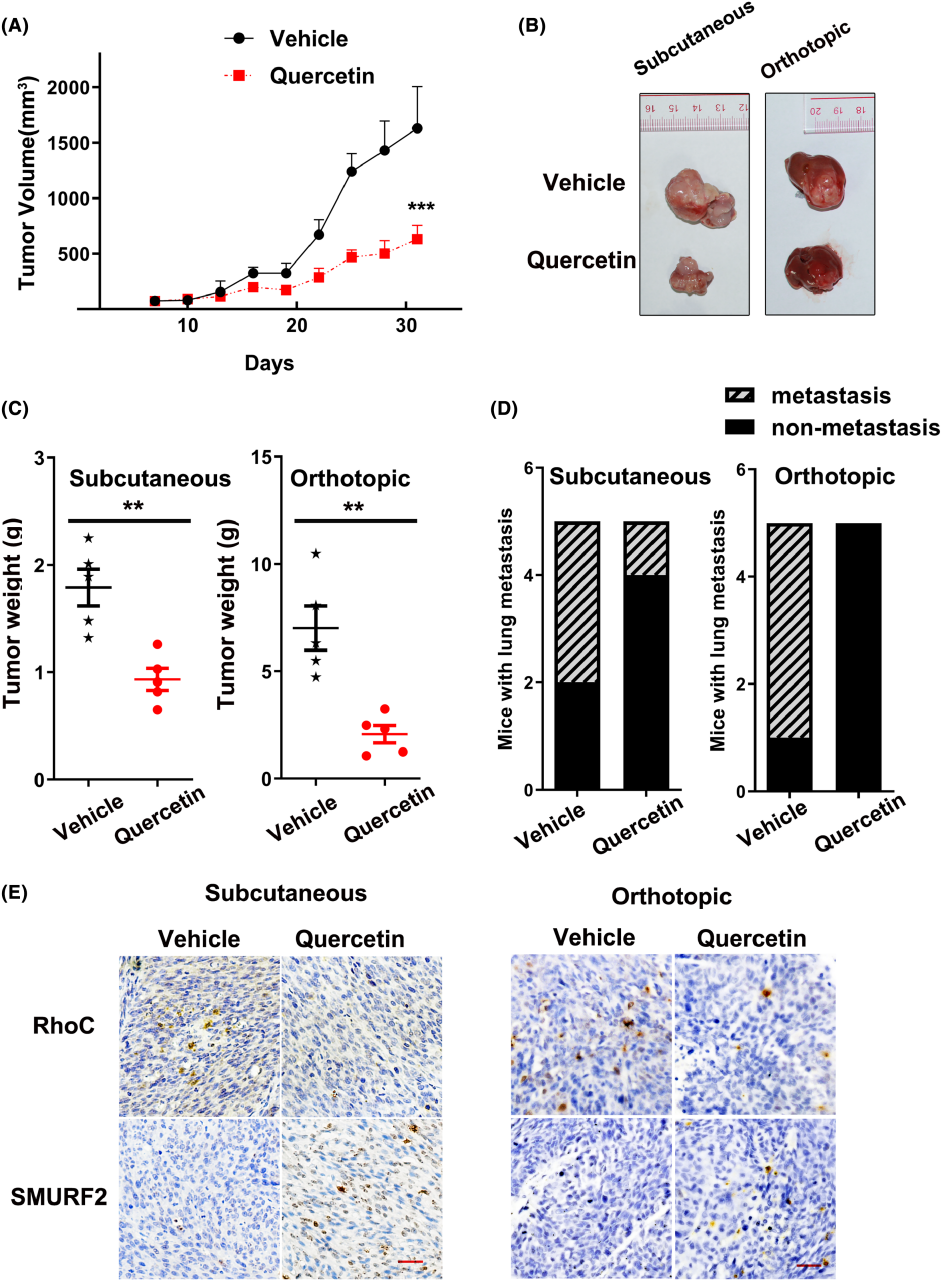
Quercetin suppresses the growth and lung metastasis of HCC in nude mice. (A) Growth curves of tumors derived from Huh7 cells treated with or without quercetin. Cells were subcutaneously injected into nude mice (*n* = 5). Tumors were isolated at the end of the experiments. (B) Representative images of tumors from each group at the experimental endpoints for the subcutaneous or orthotopic transplantation models. (C) The weight of tumor tissues was assessed in the vehicle/quercetin groups. (D) Lung metastasis of subcutaneous or orthotopic HCC in nude mice after quercetin/vehicle treatment. (E) Representative IHC staining for RhoC and SMURF2 in tumor tissues from Figure 5B, scale bars: 50 μm. **p < 0.01, ***p < 0.001.

To evaluate the in vivo effects of quercetin on the growth and metastasis of HCC, mice with subcutaneous or orthotopic Huh7 tumors were established and administered vehicle/quercetin. In the subcutaneous model, quercetin remarkably restricted tumor growth compared to that in the control group (Figure 5A), according to the tumor growth curves. At the endpoint of the experiments, the sizes and weights of tumors in the subcutaneous or orthotopic models were assessed. As shown in Figure 5B, tumor sizes were obviously smaller in the quercetin group than in the vehicle group. Moreover, the tumor weight in the quercetin group was lighter than that in the vehicle group (Figure 5C). Therefore, our data demonstrated that quercetin suppresses the growth of HCC in subcutaneous and orthotopic xenograft mice. In terms of tumor metastasis, in the subcutaneous model, only one case of lung metastasis was observed after quercetin treatment, and three cases of lung metastasis were noticed in the control group (Figure 5D). There were similar results in the orthotopic model: the control group developed many more lung metastatic foci than the quercetin group. Therefore, quercetin treatment prevents the metastasis of HCC in vivo. In conclusion, these data provide evidences that quercetin delays the progression of HCC in vivo.

### 3.6 Quercetin promotes SMURF2 expression and reduces RhoC's level in primary HCC cells and PDXs

In addition, the levels of RhoC and SMURF2 were further detected in subcutaneous and orthotopic xenograft tumor sections by IHC staining. The levels of RhoC were downregulated, while the protein level of SMURF2 was elevated in the quercetin treatment group compared with the vehicle control group (Figure 5E). The clues indicated that quercetin suppresses the metastasis of HCC by increasing SMURF2 expression and decreasing RhoC protein expression.

**FIGURE 6:**
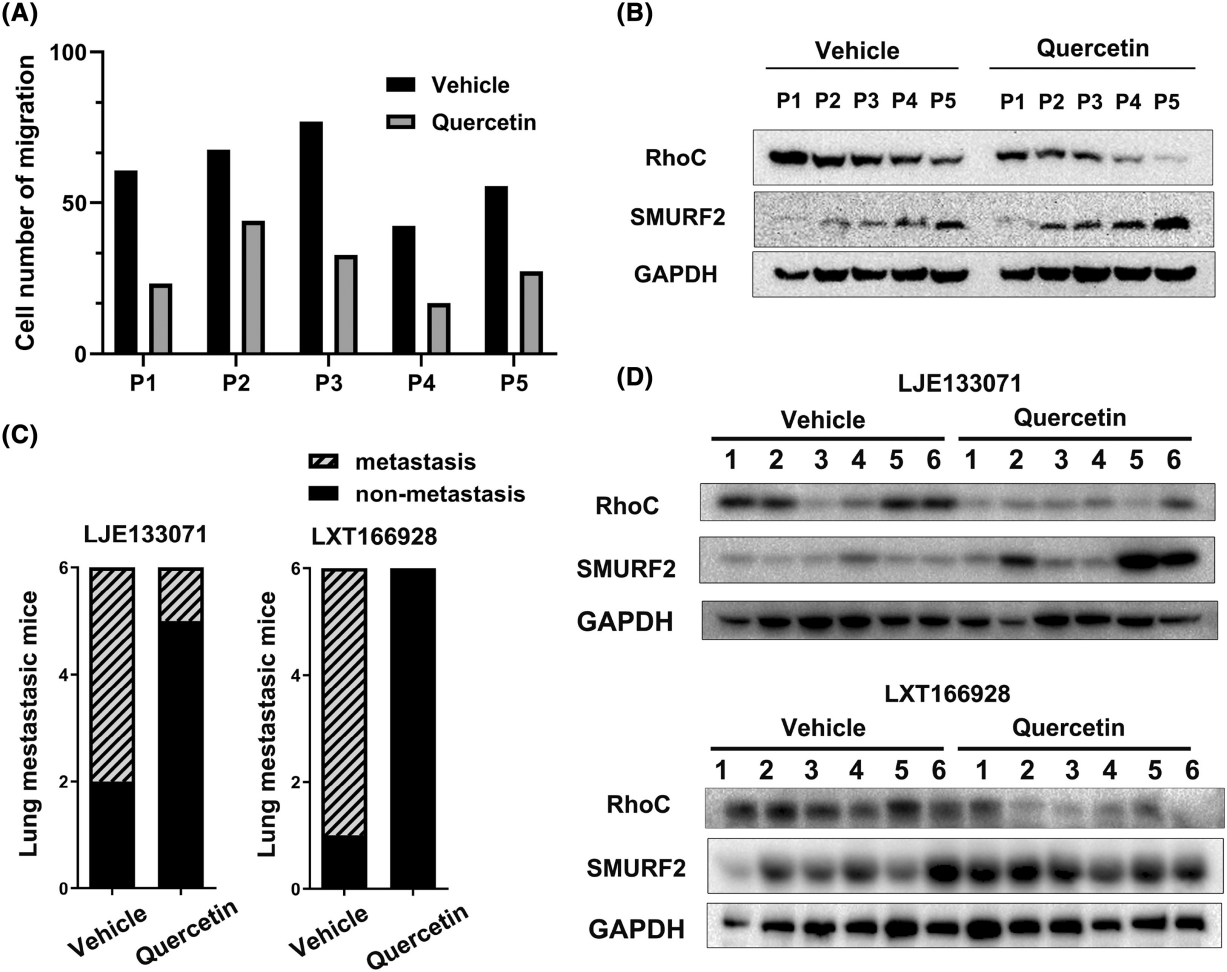
Quercetin hinders cell migration by regulating RhoC and SMURF2 levels in primary HCC cells or PDXs. Primary HCC cells isolated from five patients were incubated with or without quercetin. (A) Cell migration was evaluated in primary HCC cells. P represents patient. (B) The protein levels of RhoC and SMURF2 in primary HCC cells were determined by western blotting. (C) Lung metastasis of liver cancer in the indicated PDXs after quercetin/vehicle treatment. (D) The protein levels of RhoC and SMURF2 in PDXs were determined by western blotting. 1 ~ 6 represent tumor samples in mice.

Moreover, we studied the effects of quercetin on the migration ability of primary HCC cells derived from patient tumor samples. As indicated in Figure 6A, quercetin reduced the migration of HCC cells, suggesting its inhibitory effect on the migration ability of primary HCC cells. Additionally, in agreement with our previous descriptions in vitro, western blot analysis showed that quercetin potentiated SMURF2 expression and downregulated RhoC protein levels in primary HCC cells (Figure 6B).

Here, we established PDX models to confirm the anti‐metastatic effects of quercetin on HCC. Fresh primary tumor samples resected from HCC patients were implanted into immunocompromised mice, and the RhoC/SMURF2 protein levels were examined. We observed that quercetin hindered the lung metastasis of liver cancer in PDX model mice (Figure 6C). In addition, the western blotting results indicated that quercetin promotes the expression of SMURF2 and the degradation of the RhoC protein (Figure 6D).

## 4 DISCUSSION

SMURF2 is an E3 ubiquitin ligase that can ubiquitinate and degrade some substrate proteins.^31^ For example, SMURF2 is identified as a Smad ubiquitin ligase that participates in the ubiquitination and degradation of Smad1 and Smad2.^32^ Here, our study revealed that quercetin enhances SMURF2 expression and reduces RhoC protein levels in vitro and in vivo. Additionally, ubiquitination assays indicated that SMURF2 participates in the ubiquitination of the RhoC protein. Therefore, we inferred that SMURF2 is a RhoC ubiquitin ligase that is involved in its proteasome‐mediated degradation.

According to recent studies, the role of SMURF2 as a promoter or suppressor of tumors appears to be context dependent.^29^ Evidence has demonstrated an oncogenic role of SMURF2 in breast cancer and colorectal cancer.^32^, ^33^ However, SMURF2 knockout or heterozygous mice are prone to develop spontaneous tumors.^34^, ^35^ Additionally, aberrantly high levels of SMURF2 have been found in cancers, such as renal and prostate carcinomas.^36^, ^37^ Mechanistic studies have illustrated that SMURF2 could induce the senescence of tumor cells^38^, ^39^ and enhance the degradation of oncogenic transcription factors KLF5 and YY1.^40^, ^41^ These findings support that SMURF2 also functions as a tumor suppressor in human cancers. Our research provides new insight that SMURF2 might promote the degradation of RhoC and then inhibit invasion and migration, supporting the role of SMURF2 as a tumor suppressor.

Some oncogenic signal pathways, such as PI3K/AKT/mTOR, Wnt/β‐catenin, cMET, and IGF, play critical roles in progression of HCC and can be used for anticancer therapeutic targets.^6^ Here, our research suggested RhoC might be an effective therapeutic target for HCC. Deletion of RhoC could attenuate invasion and migration abilities of HCC without affecting cell survival (Figure S8). Moreover, we uncovered for the first time that quercetin could decrease RhoC expression level in HCC cell lines in SMURF2 dependent ubiquitination pathway. Consistently, quercetin treatment inhibits PDXs tumor growth distant invasion and metastasis. The expression level of RhoC was suppressed in quercetin treated group compared to control. Our finding provides a novel promising therapeutic approach for future clinical application of quercetin to prevent HCC metastasis and recurrence.

## AUTHOR CONTRIBUTIONS

**Chunlong Huang:** Methodology (equal); validation (equal); writing – original draft (equal). **Weihua Lai:** Conceptualization (equal); data curation (equal); validation (equal). **Shuai Mao:** Investigation (supporting); validation (supporting). **Deli Song:** Investigation (supporting); writing – review and editing (equal). **Jihong Zhang:** Conceptualization (equal); funding acquisition (equal); resources (equal). **Xiao Xiao:** Funding acquisition (lead); project administration (equal); writing – review and editing (lead).

## CONFLICT OF INTEREST STATEMENT

The authors declare that they have no competing of interests.

## ETHICS STATEMENT

This study involving animals and human tumor samples was approved by the Ethical and Welfare Committee of the First Affiliated Hospital of Sun Yat‐Sen University ([2013]94). Our research was done in agreement with local animal guidelines, ARRIVE guidelines and the Declaration of Helsinki.

## INFORMED CONSENT

Written informed consent was obtained from HCC patients.

## ANIMAL STUDIES

All animal experiments were performed incompliance with the animal welfare of the First Affiliated Hospital of Sun Yat‐Sen University.

## Supporting information


Data S1.

## Data Availability

The data that support the findings of this study are available from the corresponding author upon reasonable request.
