# Supplementary material for: Quercetin‐induced degradation of RhoC suppresses hepatocellular carcinoma invasion and metastasis
Source: Cancer Med. 2024 Mar 8;13(4):e7082. doi: 10.1002/cam4.7082 (PMC10923047; doi:10.1002/cam4.7082)
Supplement: Supplementary file 1 — Data S1. [file CAM4-13-e7082-s001.docx]

**Table S1. Predicted E3 ligases for RhoC**

| **Rank** | **Gene symbol** | **Gene Full Name** | **Score** |
| --- | --- | --- | --- |
| 1 | SMURF1 | SMAD specific E3 ubiquitin protein ligase 1 | 0.799 |
| 2 | NEDD4 | developmentally downregulated protein 4 | 0.766 |
| 3 | SMURF2 | SMAD specific E3 ubiquitin protein ligase 2 | 0.718 |
| 4 | UBE3C | ubiquitin protein ligase E3C | 0.716 |
| 5 | CBL | Cbl proto-oncogene | 0.681 |
| 6 | UBR5 | ubiquitin protein ligase E3 component n-recognin 5 | 0.670 |
| 7 | WWP1 | WW domain containing E3 ubiquitin protein ligase 1 | 0.670 |
| 8 | WWP2 | WW domain containing E3 ubiquitin protein ligase 2 | 0.669 |
| 9 | SYTL4 | synaptotagmin like 4 | 0.664 |
| 10 | HUWE1 | HECT, UBA and WWE domain containing E3 ubiquitin protein ligase 1 | 0.660 |





**Fig. S1 The chemical structure of quercetin.**


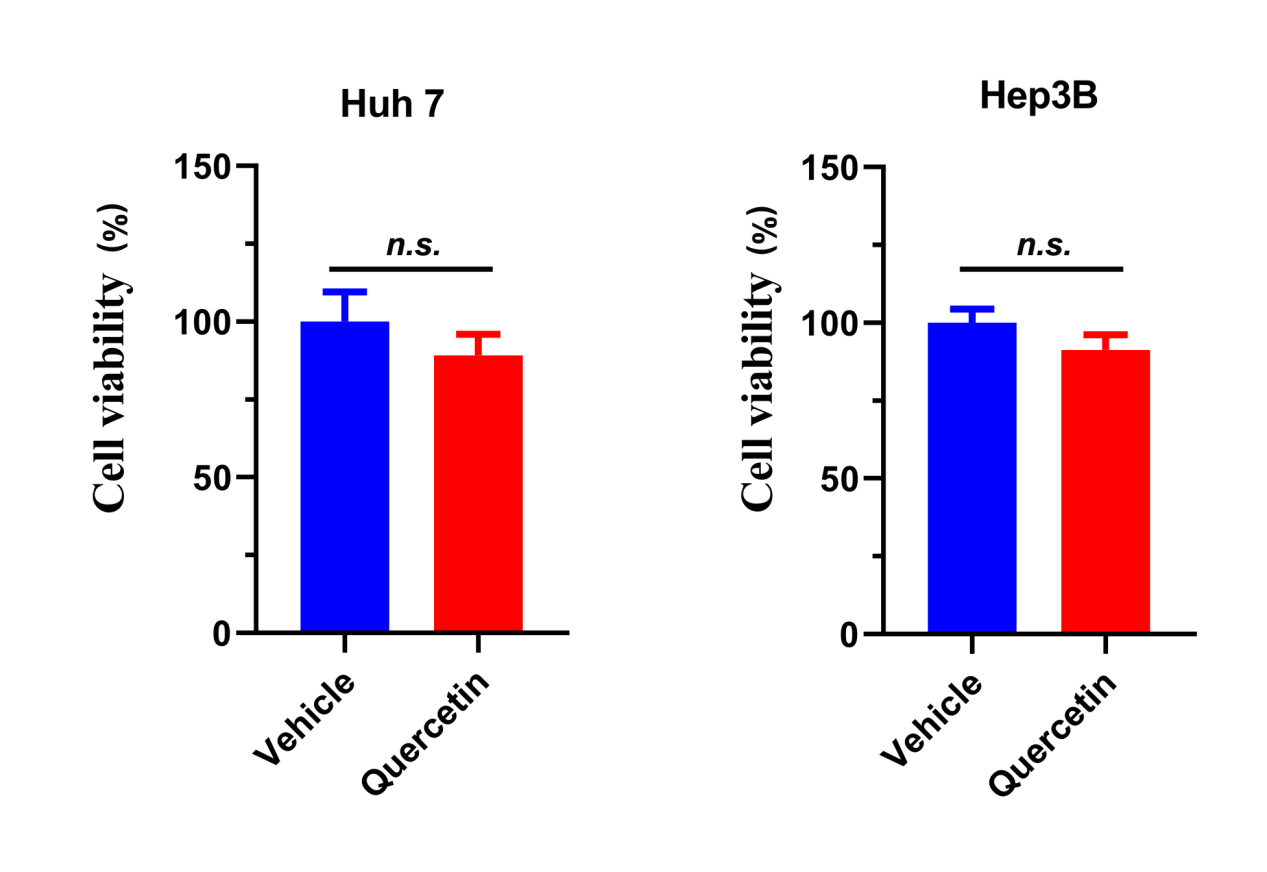


**Fig. S2 The effect of quercetin on cell viability of HCC.** Huh7 (left) and Hep3B (right) cells were treated with quercetin. . Then cell viability was detected by CCK8 assay after 48h.


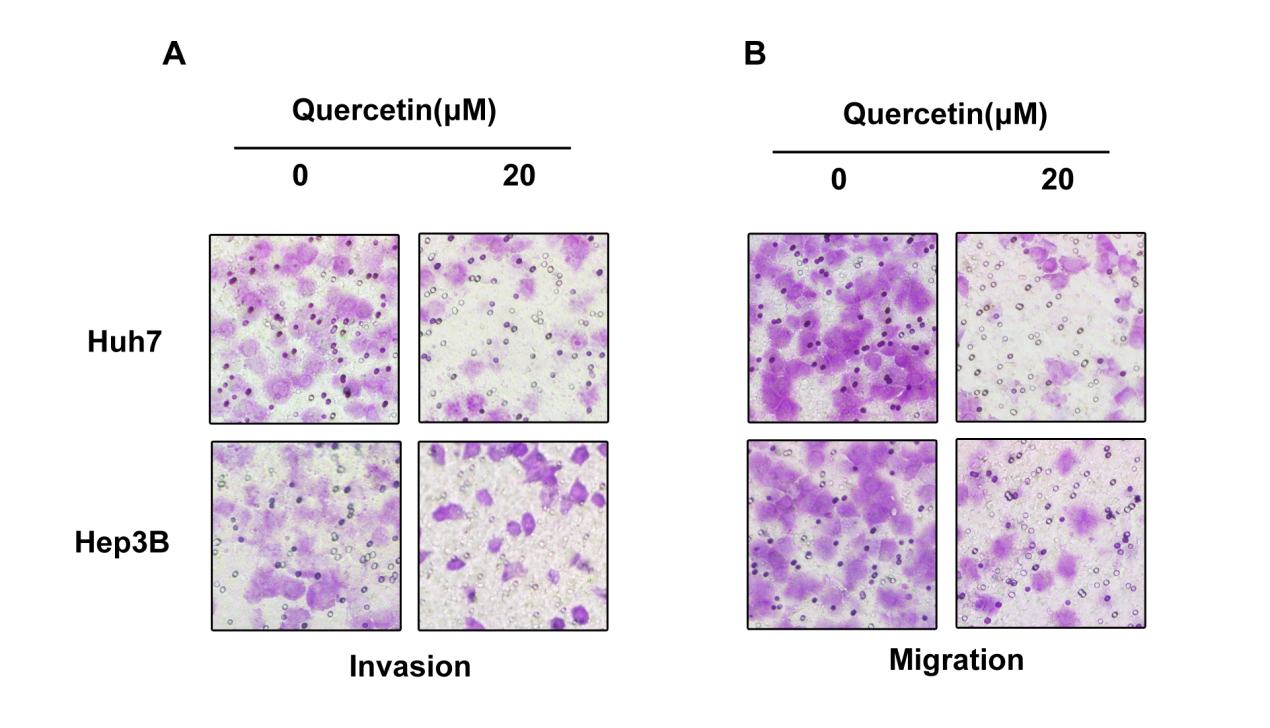


**Fig. S3 The effect of quercetin on cell invasion and migration of HCC.** Huh7 (up) and Hep3B (down) cells were treated with indicated concentrations of quercetin for 24h. A, The invasion ability was accessed via microscope images. B, The migration ability was evaluated via microscope images.

**
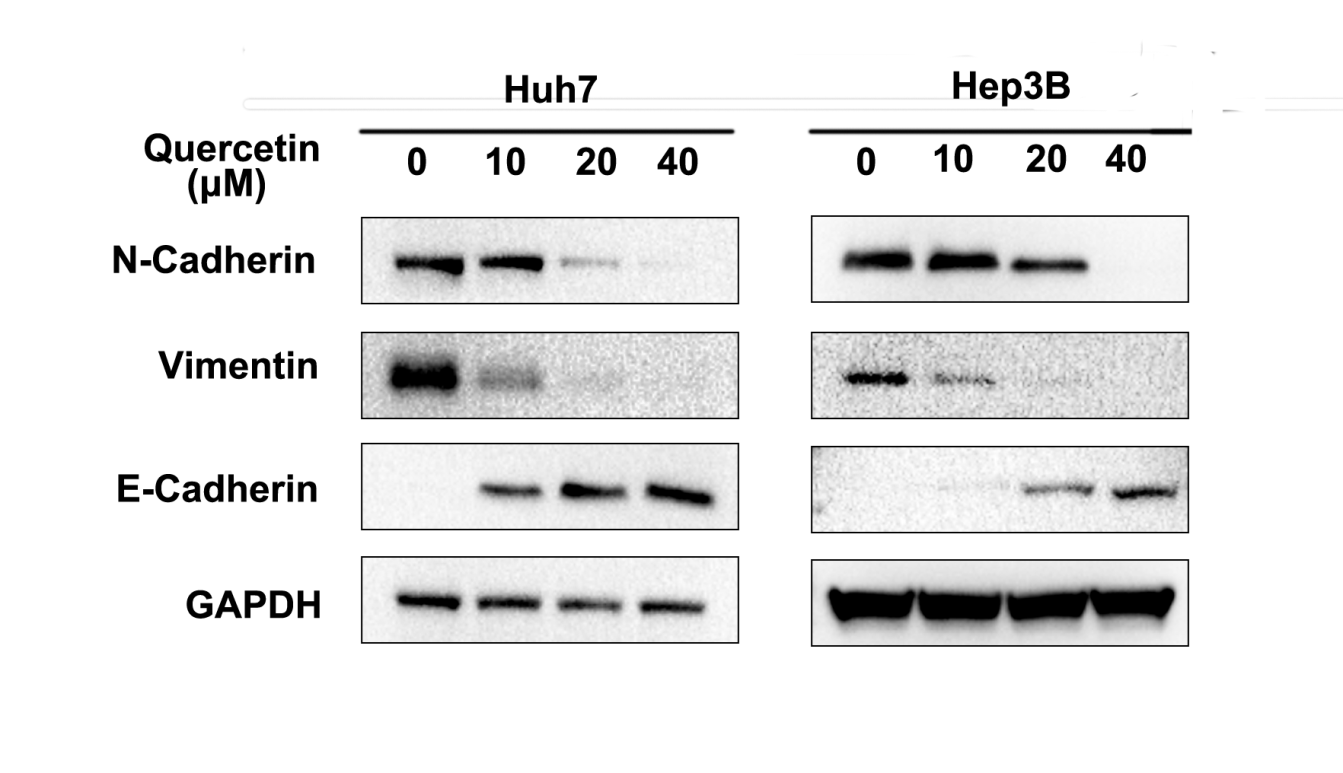
**

**Fig. S4 Quercetin impairs epithelial-mesenchymal transition of HCC cells**


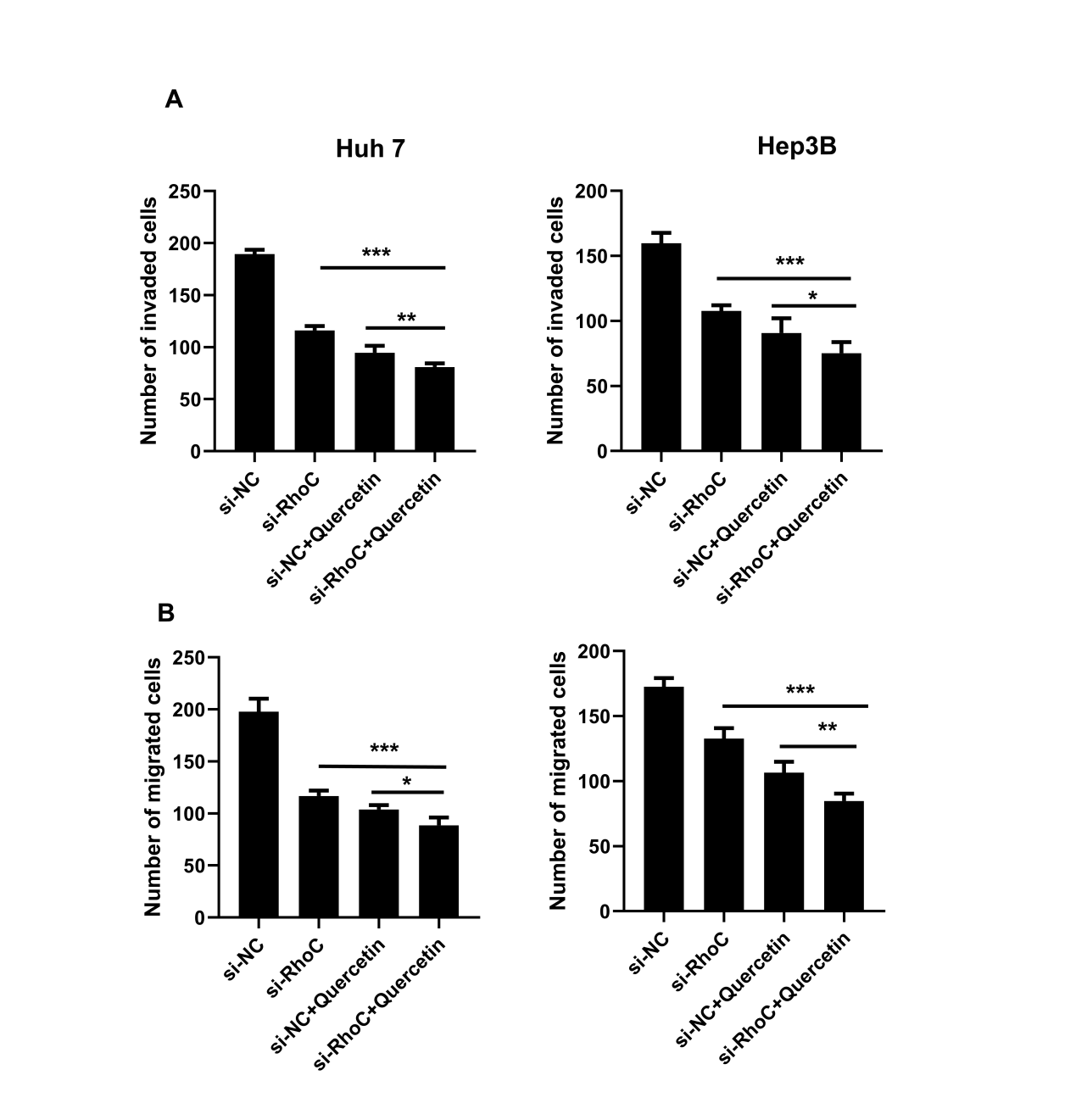


**Fig. S5 Quercetin attenuates the invasion and migration of HCC cells via RhoC.** Huh7 (left) and Hep3B (right) cells were treated with indicated treatment: siRNA negative control, siRNA RhoC, quercetin. A, The number of invaded cells was counted. B, The number of migrated cells was counted. **P*<0.05, ***P*<0.01, ****P*<0.001.


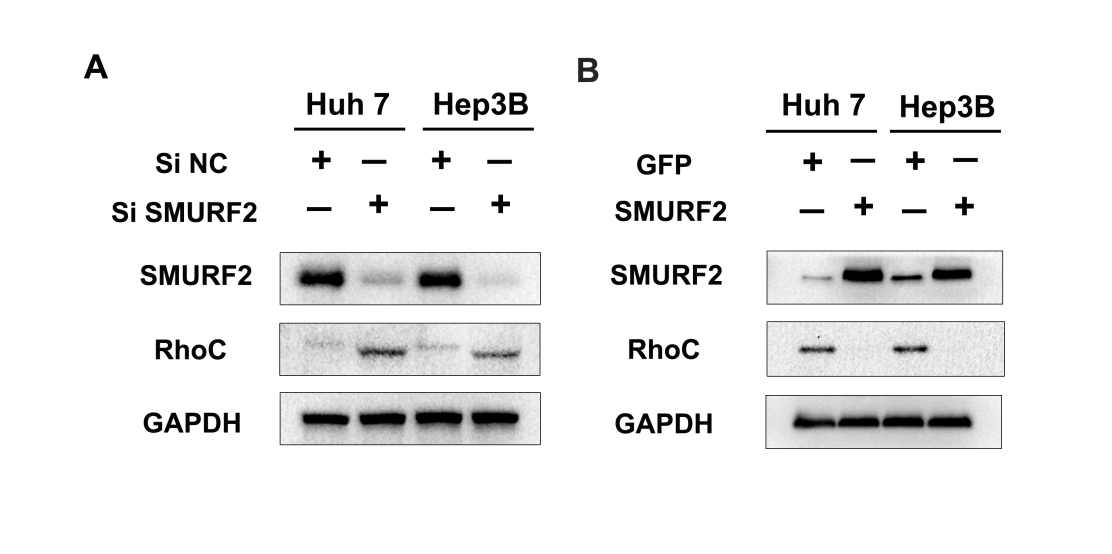


**Fig. S6 SMURF2 negatively regulates the protein level of RhoC.** A, Huh7 (left) and Hep3B (right) cells were incubated with siRNA Negative Control or SMURF2. B, Huh7 (left) and Hep3B (right) cells were transfected with plasmids expressing GFP (negative control) or SMURF2.


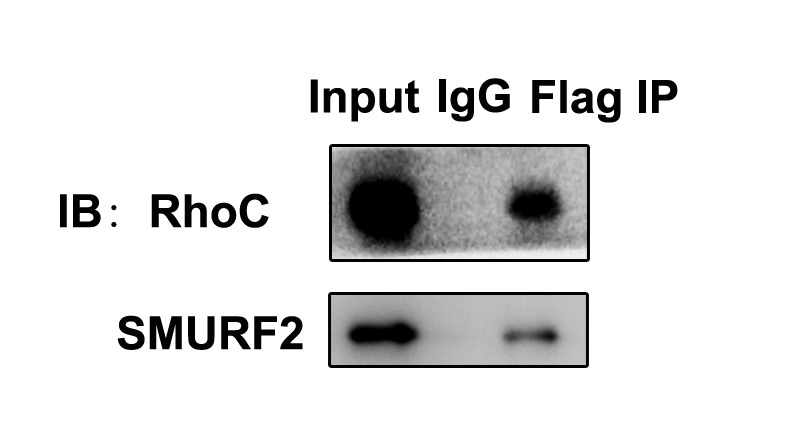


**Fig.S7 RhoC interacts with SMURF2.** 293T cells were transfected with Flag-tagged constitutively RhoC expression vectors, and subjected to immunoprecipitation (IP) with IgG or an anti-Flag antibody. Input lysates and anti-Flag immunoprecipitation eluates were subsequently detected by immunoblot (IB) using RhoC and SMURF2 antibody.


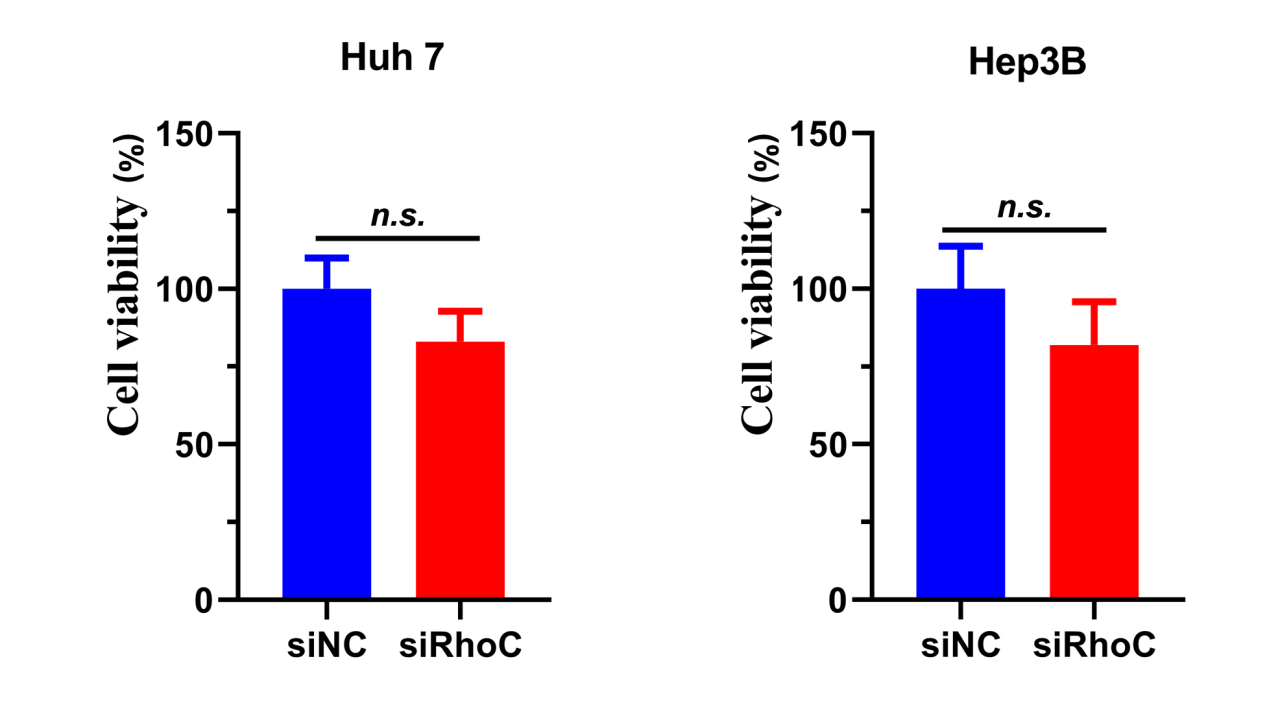


**Fig. S8 The influence of RhoC on cell viability of HCC.** Huh7 (left) and Hep3B (right) cells were incubated with siRNA NC or RhoC. Then cell viability was determined by CCK8 assay after 48h.
